# Lung Tissue Resident Memory T-Cells in the Immune Response to *Mycobacterium tuberculosis*

**DOI:** 10.3389/fimmu.2019.00992

**Published:** 2019-05-03

**Authors:** Paul Ogongo, James Zachary Porterfield, Alasdair Leslie

**Affiliations:** ^1^Africa Health Research Institute, Durban, South Africa; ^2^School of Laboratory Medicine and Medical Sciences, University of KwaZulu-Natal, Durban, South Africa; ^3^Institute of Primate Research, National Museums of Kenya, Nairobi, Kenya; ^4^College of Health Sciences, University of KwaZulu-Natal, Durban, South Africa; ^5^Yale School of Public Health, Yale University, New Haven, CT, United States; ^6^Department of Infection and Immunity, University College London, London, United Kingdom

**Keywords:** tuberculosis, T-cell, tissue resident memory, vaccine, lung

## Abstract

Despite widespread BCG vaccination and effective anti-TB drugs, Tuberculosis (TB) remains the leading cause of death from an infectious agent worldwide. Several recent publications give reasons to be optimistic about the possibility of a more effective vaccine, but the only full-scale clinical trial conducted failed to show protection above BCG. The immunogenicity of vaccines in humans is primarily evaluated by the systemic immune responses they generate, despite the fact that a correlation between these responses and protection from TB disease has not been demonstrated. A novel approach to tackling this problem is to study the local immune responses that occur at the site of TB infection in the human lung, rather than those detectable in blood. There is a growing understanding that pathogen specific T-cell immunity can be highly localized at the site of infection, due to the existence of tissue resident memory T-cells (Trm). Notably, these cells do not recirculate in the blood and thus may not be represented in studies of the systemic immune response. Here, we review the potential role of Trms as a component of the TB immune response and discuss how a better understanding of this response could be harnessed to improve TB vaccine efficacy.

## Introduction

Despite nearly a century of BCG vaccination and the development of highly effective anti-TB drugs, Tuberculosis (TB), remains the main cause of death from a single infectious agent (1). There were approximately 6.3 million new cases of TB and 1.6 million deaths from TB in 2016, and it is estimated that a quarter of the world population is infected with *Mycobacterium tuberculosis (Mtb)*. However, only 5–10% of these individuals will develop active disease during their lifetime (2), suggesting that the immune system is highly effective at containing *Mtb* infection in most people.

The *Mycobacterium bovis* Bacille-Calmette-Guérin (BCG) vaccine is currently the only approved vaccine for immunization against TB. It is an attenuated strain of *M. bovis* that provides highly efficient protection against TB in children (3). The WHO currently recommends that BCG vaccination be given soon after birth in all countries where risk of TB infection is high, and more than 4 billion individuals have been vaccinated to date (4, 5). However, the vaccine has shown highly variable efficacy and performs poorly in adults and in the developing world (3). Moreover, the protection offered to children is not lifelong, tending to last for up to 20 years, rendering them susceptible to TB acquisition at an age when TB incidence is increased (6). As a result, it has been estimated that, despite it's widespread use, BCG may avert only ~5% of vaccine preventable deaths (7). Therefore, a new vaccine or vaccination strategy against tuberculosis that yields improved protective immunity in all age groups is necessary to contain the spread of tuberculosis and reach the targets of the End-TB Strategy by 2035 as set out by the World Health Organization.

T-lymphocytes have been shown to be critical for the prevention of primary disease from initial *Mtb* infection, and also the development of post primary TB once latent infection has been established (8–13). Consequently, the loss of CD4 T-cells through HIV-infection in humans, or via experimental depletion in mice and non-human primates, greatly exacerbates TB susceptibility and reactivation of latent infection (11, 14, 15). Following antigen encounter in lymph nodes (LNs), naïve T-cells undergo rapid proliferation, giving rise to differentiated effector T-cells, and long lived memory T-cells that are distributed more broadly throughout the body (16). On re-exposure, memory T-cells are able to mount a more rapid and robust response to the antigen (17, 18), which is the basis of vaccine efficacy. There is growing evidence that this memory response is most effective if it is positioned at the site of pathogen infection. This is possible as a subset of memory cells, called Tissue Resident Memory (Trm) cells (19), can persist in tissue for an extended period, without recirculating in blood, ready to rapidly respond to a new infection. HIV infection in humanized mice and SIV infection in non-human primates were recently shown to preferentially deplete Trm CD4 T-cells from the lung parenchyma, compared to both blood and the alveolar space (20). In humans, HIV infection is associated not only with a greatly increased risk of active TB but also with a greater risk of disseminated infection. Therefore, lung Trm are likely to be essential for controlling pulmonary TB in humans.

Recent exciting data, investigating novel vaccination routes in animal models, suggests that BCG activity is improved when it induces such a memory T-cell response within the lung (21, 22). Here we briefly review the biology of Trms, examine the evidence that they play a significant role in the immune response to *Mtb* infection and discuss how they might be harnessed to improve vaccine efficiency against this deadly infection.

## Tissue Resident Memory T-Cells (Trms)

Protective T-cell responses should be rapid and robust at the primary site of infection and should confer durable protection (23–26). Since *Mtb* is transmitted primarily as an aerosol, the initial immune response occurs at the pulmonary mucosal surfaces (27–30). Trms are a recently described population of T-cells that have functional and transcriptional similarities to central and effector memory T-cells, but which remain embedded in tissue site for prolonged periods (31). As a result, they are positioned to function as a first line of defense against subsequent infection in these tissues.

During an infection, activated T-cells migrate into inflamed tissue under the guidance of chemokines, integrins, and adhesion molecules (32). T-cells that encounter their specific antigen at the infection site expand and receive tissue-specific cues, which influence their function and memory potential (33). Following pathogen clearance, some of these effector memory T-cells remain within the tissue site and form a non-recirculating Trm population which remains *in situ* to protect against local pathogen re-exposure. Trms have been described in many non-lymphoid tissues, including the skin, gut, female genital tract and the lungs; typically, at the sites of initial infectious exposure and often at a mucosal surface. As a result of this positioning, Trms can play a critical role in subsequent immune challenge by accelerating clearance of local reinfections at these mucosal surfaces (25, 34–37). A direct consequence of this tissue residency, is that Trms are typically absent from blood (38, 39), which can have profound implications for their study in humans, in whom peripheral blood mononuclear cells (PBMCs), are often the main substrate for studying immune responses.

Trm cells vary in phenotype and function, depending on the tissue they reside in (17, 21, 40, 41). The study of Trm is complicated by the need to differentiate cells truly isolated from the parenchyma from cells contained within the lung vasculature. The highly vascularized nature of lung tissue means that these cells are abundant and are not possible to eliminate during sample processing. Much of the biology of this subset has been worked out in mice and has relied on several novel approaches which get round this problem; notably, parabiosis, intravenous labeling of circulating cells just prior to sacrifice to distinguish cells in circulation (labeled) to those in tissue (unlabeled), and the use of Fingolimod (FTY720), a drug which prevents T-cell egress from lymph nodes and thus blocks new recruitment of memory cells into tissue sites (33, 42–48). Although these techniques are not applicable to humans, they have confirmed that many of the cell surface markers used in animal models are shared in humans, notably high CD69 expression together with CD103 (26, 49). While CD69 is expressed by the majority of Trms, CD103 is mostly expressed on a subset of Trms, primarily CD8^+^ T-cells (26). Numerous other cell surface markers have been associated with Trm cells, including the upregulation of CD49a (an adhesion molecule), CXCR6, CD101, PD-1, and loss of CD62L and the chemokine receptor CX3CR1. Although there does not appear to be lung specific Trm marker(s) *per se*, Table 1 highlights those that have been associated with T-cell residency within the lung. Many of these markers have been used to distinguish between lung parenchymal and intravascular T-cells. However, it is important to note that there are almost certainly exceptions to some or all of these markers in the lung, as in other tissues, and work is needed to improve and refine them.

**Table 1:** Cell surface markers of lung Trms in humans validated in animal models.

**Marker**	**Tissues studied**	**Pathogen or condition studied**	**Reference**
CD103(integrin αE) Functions: • Combines with integrin β7 to form αEβ7 heterodimer • Involved in leukocyte retention and activation	Upregulated in: Skin, Lung, FRT, Kidney, Gut	*M. tuberculosis*, Influenza, HSV, LCMV,	(16, 21, 50, 51)
CD11a (ITGAL-1) Functions: • Combines with β2 to form LFA-1 • Involved in adhesion and co-stimulation	Upregulated in: Lung, Skin	Influenza, Asthma, *Leishmania major*	(52–54)
CD49a (VLA-1α: α1 integrin) Functions: • Combines with integrin β1 subunit to form α1β1 heterodimer • Involved in adhesion	Upregulated in: Lung	*M. tuberculosis*, Influenza	(21, 55)
VLA-4 (α4β1: integrin dimer composed of α4 (CD49d) subunit and β1(CD29) subunit) Functions: • Involved in adhesion, cell migration, and activation	Upregulated in: Lung	*M. tuberculosis*	(56)
CD69 Function: • Binds to and downregulates S1PR1 • Involved in lymphocyte tissue retention	Upregulated in: Skin, Lung, FRT, Kidney, Gut	*M. tuberculosis*, Influenza, HSV, LCMV,	(19, 57–60)
CD101 Functions: • T-cell activation and proliferation	Upregulated in: Skin, intestines, Liver, Lung	*Plasmodium*	(61)
CD44 Functions: • Leukocyte rolling and homing	Upregulated in: Lung	*M. tuberculosis*, Influenza	(19, 39)
CD62L Functions: • Leukocyte rolling and homing	Downregulated in: Gut, Skin, Lung, Lymph Node	HIV, Vaccinia virus	(62, 63)

*ITGAL, Integrin alpha L 1; LFA, lymphocyte antigen associated-antigen 1; VLA, very late antigen; FRT, female genital tract; HSV, herpes simplex virus; LCMV, lymphocytic choriomeningitis virus; HIV, human immunodeficiency virus*.

## Formation and Maintenance of Tissue Resident Memory T-Cells

Understanding the signals driving the formation and maintenance of Trm cells is crucial if they are to be exploited by novel vaccination strategies. Trm cells derive from precursors entering tissues during the effector phase of immune responses and remain positioned within these compartments (35, 64). For this to occur, they must adapt to local survival cues, resist shedding into the lumen at mucosal epithelial surfaces, and ignore egress signals (18, 32). The transition of recruited T-cells to Trm cells under normal conditions generally requires simultaneous tissue damage and TCR (T cell receptor) signaling on antigen exposure (55, 65, 66). Thus, whilst on-going inflammation may recruit non-specific T-cells from circulation, only those that encounter their cognate antigen should set up tissue residency. However, this is not always the case (50, 67) and may vary according to location. Flu-specific Trms in the upper respiratory tract, but not in lung tissue, for example, can develop independently of local cognate antigen recognition (51), possibly as a consequence of localized production of cytokines such as IL-15 (68, 69). Nonetheless, any infection in non-lymphoid tissue that is antigenic and causes pathology has the potential to establish a Trm population at that site.

One consequence of TCR activation in general is temporary upregulation of the canonical Trm marker surface CD69 (70–72), which interacts with sphingosine-1-phosphate receptor 1 (SIPR1) and downregulates its expression (73–77). This is probably a key mechanism for the generation and maintenance of Trms as it prevents cells from following the S1P chemokine gradient back into circulation, and therefore results in prolonged retention and local memory formation (57). However, it is not clear how CD69 expression is sustained in cells that become Trm, and whether all CD69 positive T-cells identified in tissue at any given time are actually Trm. Indeed, although it is widely accepted as a good marker, there is some evidence that not all Trm constitutively express CD69 (35). Another molecule that may help retain Trm in tissue is the integrin αE (CD103) (78), which forms a heterodimer with the integrin β7 and binds E-cadherin (23), a glycoprotein constitutively expressed by epithelial cells. Therefore, CD103 is thought to be particularly important for retaining Trm at the epithelial surfaces of mucosal barriers such as the lung. Other integrins and key modulators thought to be involved in retention of Trm and their function in tissue sites are highlighted in Table 1.

The mechanistic details of how Trm responsiveness and longevity is maintained remains an area of much on-going research. In central memory T-cell populations, enhanced lifespan, and proliferative capacity is associated with a shift in metabolism toward endogenous lipids and oxidative phosphorylation (79). Similarly, skin CD8^+^ Trm appear to rely on fatty acid β-oxidation to support their long-term survival, although via exogenous lipid uptake (80). In addition, RNA sequencing of Trms in both animal models and humans shows there is a distinct transcriptional profile associated with tissue residency (26, 81, 82). Thus, transcriptional and metabolic reprogramming probably underlie the formation and maintenance of Trm within their tissue niches. Two recent publications have elegantly shed light on the dynamics of Trm cell formation and maintenance *in situ*. First, intra-vital imaging of the female genital tract revealed that CD8^+^ Trm cells continually patrol the tissue, but pause and rapidly expand after LCMV challenge (58). These data are important, as they remove the image of Trm as a sedentary cell waiting to encounter its pathogen, and provide a clear mechanism by which a relatively small number of Trm may effectively protect a large organ or mucosal barrier. In this study, LCMV infection also triggered the recruitment of recirculating memory T-cells, but the expansion of pre-existing Trms was independent of this process and far outweighed that of memory cells recruited from circulation (58). In a separate study, Park et al. demonstrated that newly recruited T-cells could establish a Trm population in the skin without displacement of the pre-existing Trm pool. This is also important as it demonstrates that multiple Trm cell specificities can be stably maintained within the same tissue (83). Together these studies provide a clear mechanistic rationale for how vaccine induced Trms against *Mtb* could be established against the backdrop of existing Trm to protect a large organ such as the lung (84).

From the perspective of *Mtb* research, it is unfortunate that the majority of work on the biology of Trm has focused on tissues other than the lung. Moreover, there is evidence that Trm populations are not as stable in lung tissue as they are in organs such as the skin (85, 86). However, observations of long-lived Trms against respiratory pathogens in the lungs of both mice, non-human primates and humans have been made (38, 84, 87). Although more work is needed, it seems reasonable to assume that life time exposure to lung pathogens, should build up a repertoire of Trms that patrol the lung and are poised to rapidly respond to new re-infection or re-activation of latent disease, as may be the case in TB. Indeed, regularly low level exposure to *Mtb* antigen, either through transitory blip in latency (88), or exposure to *Mtb* or other environmental mycobacteria might be expected to facilitate TB-specific Trm maintenance in the human lung.

## Trafficking of T-Cells to the Lung During Tuberculosis Infection

**Figure 1:**
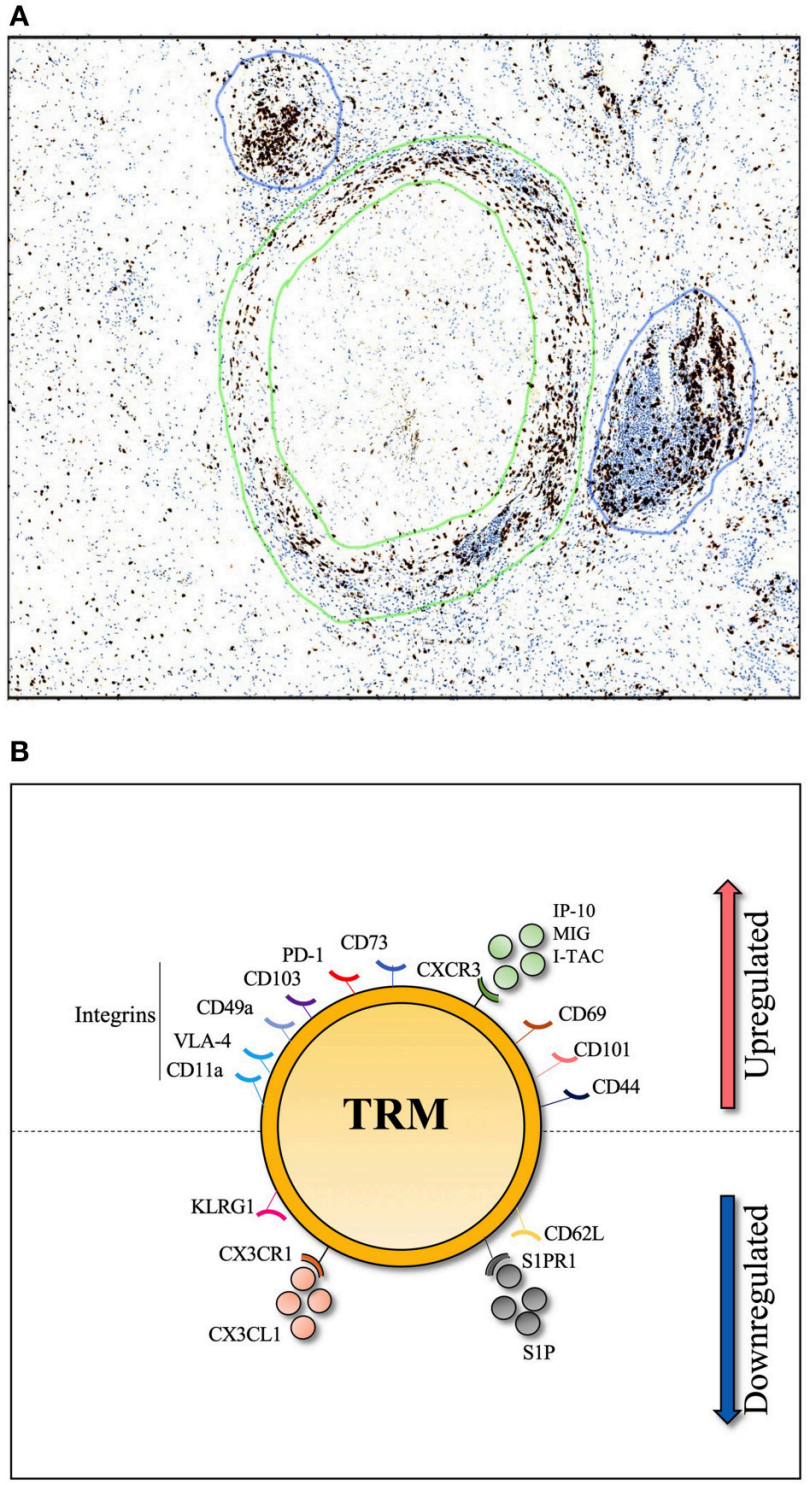
Histological section of human TB lung granuloma. **(A)** 4μm section cut from a TB infected human lung removed by surgical resection to treat TB sequelae. Lymphocyte cuff of CD3 positive T-cells (stained in brown and shown by green circles) surround a core of CD68 positive macrophages, stained in a serial histology section (not shown), granuloma associated lymphocyte follicles indicated by blue circles. **(B)** Markers associated with lung Trm including integrins (CD11a, VLA-4, CD49a, and CD103), cell surface markers (PD-1, CD44, CD101, and CD69), chemokine receptor (CXCR3) and CD73 which is important for lymphocyte binding to the endothelium. To resist tissue exit cues, lung Trm cells downregulate expression of surface markers (CD62L, KLRG-1) and chemokine receptors (CX3CR1, S1PR1).

Direct contact between CD4^+^ T-cells and antigen presenting cells is required for a robust T-cell response to *Mtb* infection (89). T-cells within the lung can be divided into parenchymal and intravascular subsets. In TB infected mice, T-cells that cross the lung vasculature into the parenchyma express CXCR3 and are negative for Killer Cell Lectin Like Receptor G1 (KLRG1), while intravascular CD4^+^ T-cells express CX3CR1 and expressed a more terminally differentiated, KLRG1^hi^ /T-bet^hi^, phenotype (19). Importantly, when adoptively transferred to naïve mice, CXCR3^+^ KLRG1^−^ CD4^+^ T-cells from the lung parenchyma of TB infected mice provided better immune protection than their blood counterpart (19, 90). This suggests that parenchyma derived CD4^+^ T-cells express the markers that allow them to home directly back to the lung, and contact *Mtb* infected macrophages. Upregulation of CXCR3 is likely to be key, as increased levels of CXCR3 ligands, such as IP-10, in the lung are required for T-cell lung recruitment (91). Indeed IP-10, along with other CXCR3 ligands (MIG and I-TAC) are all expressed in the granulomas of TB-infected lungs and contribute to their formation (92). This presents a model whereby, upon *Mtb* infection, primed T-cells are recruited to the lung parenchyma and are positioned within granulomas along a chemokine gradient, in a CXCR3-dependent manner (19, 93). A combination of TCR triggering and signaling from tissue damage should provide the conditions to establish a population of *Mtb* specific Trms. The classic granuloma structure is defined by a “cuff” of T-cells surrounding a core of infected phagocytes. Moreover, granuloma are frequently associated with T-cell aggregates in the form of inducible bronchioalveolar lymphoid tissue (iBALT), which are thought to be protective in TB infection (94, 95) as shown in Figure 1. Whether these T-cells are TB-specific Trm cells is not currently known, but, given these conditions, it appears biologically plausible.

## The Role of Tissue Resident Memory T-Cells in Lung Infections

There is a growing body of evidence that Trm cells play a central role in many different pathogenic infections (19, 64, 96); including several studies demonstrating the importance of tissue localized T-cells in coordinating host lung immunity (34, 46, 97–99). Respiratory viral infections, for example, are known to induce memory T-cells that persist in lung tissue and airways following infection clearance (38, 39, 87). In an influenza challenge model, mice that received adoptive transfer of lung memory CD4^+^ T-cells exhibited only minor symptoms and complete lung viral clearance by day 8 post infection, compared to naïve mice or those receiving spleen memory CD4^+^ T-cells, in whom the infection was lethal (38). Laidlaw et al. also observed a protective effect of transferring lung CD8^+^ T-cells prior to flu challenge (100), and in addition showed the importance of CD4^+^ T-cells in the generation of functional CD8^+^. Depletion of CD4^+^ T-cell prior to infection, did not affect the frequency of flu-specific CD8^+^ T-cells in the lung parenchyma, but these cells had reduced expression of the Trm markers CD103, CD69, and CXCR3. Importantly, although isolated from the lung parenchyma, these CD8^+^ T-cells did not readily localize to the airway epithelium when adoptively transferred, and had a diminished protective effect, compared to CD8^+^ T-cells generated in the presence of CD4^+^ T-cell help in the lung (100). Subsequently, the antigen specificity of lung Trm was convincingly demonstrated by adoptive transfer of Trm cells from mice infected with either Sendai virus or influenza virus to naïve hosts, who were then challenged with one or the other of these viral infection (101). Both Trms expressed high levels of CXCR3 and homed effectively to the lung, but protection was only conferred to the recipient mice challenged with the appropriate virus; i.e., Trm cells from Sendai infected mice provided protection from Sendai virus but not influenza. In addition, the protective phenotype was not seen in IFN-γ knock-out mice. Interestingly, the Trm in this study were isolated from bronchoalveolar lavage (BAL) and not from homogenized lung. Recently published work has suggested that upper airway Trms may represent a distinct population from those in the lung parenchyma, as they develop under different circumstances (51). Here, vaccination of the upper respiratory tract induced Trm that appeared to be longer lived than those induced by vaccine delivered deeper into the lung, and were more effective at blocking transmission of influenza to the lower respiratory tract. This is important to bear in mind as, for practical reasons, lung Trm in humans and non-human primates are often studied in BAL fluid. Nonetheless, the same group subsequently identified influenza-specific CD8^+^ lung Trm in excised human lung tissue (59). The expansion of both airway and lung CD8^+^ Trms via intra nasal dosing with Flu antigen and a CpG adjuvant, was found to protect mice from subsequent challenge compared to mice in whom these long lived Trms were not generated (55). Importantly, administration via the intramuscular or intraperitoneal route were equally effective at generating influenza-specific memory CD8^+^ T-cells in the lung vasculature or spleen, but failed to generate Trms in the lung parenchyma or airways and failed to protect the mice from subsequent viral challenge.

Similar observations have been made with respiratory syncytial virus (RSV), another important pulmonary viral infection. Mice infected with this virus develop antigen specific CD8^+^ Trms in the lungs and airways, and intranasal transfer of these cells to naïve mice protects them from subsequent infection (102). Here again, although intramuscular DNA immunization induced RSV specific CD8^+^ T-cells, it failed to induce sufficient lung Trms as only 10% of RSV specific CD8^+^ T-cells had a Trm phenotype. Interestingly, these non-Trm cells were actually immunopathogenic, as mice vaccinated via this route did worse than those who had not been vaccinated at all (102). Using CMV vectors, Morabito et al. were able to generate RSV-specific CD8^+^ Trms via intranasal vaccination that protected mice from subsequent infection, but not via the intraperitoneal route (103). Moreover, by using FTY720, a drug that sequesters T-cells within lymph nodes, they showed that these lung Trms were sufficient for protection and additional recruitment of memory cells had no impact. Finally, Gilchuk et al. showed that intranasal administration of engineered recombinant vaccinia virus proteins, generated lung CD8^+^ Trms that protected from subsequent lethal vaccinia infection, while the same vaccine given intraperitoneally did not (104). Here protection was linked specifically to CXCR3^lo^ CD8^+^ Trms located in the pulmonary interstitium, and mice depleted of these cells via intranasal administration of anti-CD8 antibodies rapidly succumbed to infection.

These studies demonstrate the importance of lung Trm in protection from viral pathogens, and suggest that they can be induced by an appropriate vaccination strategy. The involvement of Trms in other pulmonary infection, such as bacterial and helminthic, have been less well studied but reports of their protective effect do exist. Progressive acquisition of immunity to microbes that frequently cause pneumonia in early life was recently demonstrated by infecting mice with mis-matched serotypes of *Streptococcus pneumoniae* (105). Prior infection with mildly pathogenic strains provided protection against subsequent highly virulent pneumococcal infection through the formation of IL-17 producing CD4^+^ Trms. Interestingly, these were only formed in the lung lobe that was initially infected, and only these lobes were protected from subsequent infection with the more virulent strain. These data not only confirm the potential of Trms to protect the lung from bacterial pathogens, but also demonstrate they can be highly localized within this organ. Pathogen specific Trms have been observed to respond more rapidly than circulating T-cells in mice experimentally infected with the gram-negative bacteria *Bordetella pertussis* (106), although the importance of these cells was not formally demonstrated. Lastly, in a helminth model, using FTY720 to prevent T-cell recruitment from lymph nodes, it was found that lung resident T-cells were sufficient for effective recall immunity to *Nippostrongylus brasiliensis* infection (107). Taken together, these studies show that, as in other organs, lung and airway Trms, both CD4 and CD8, form an important part of the immune response to a wide variety of pathogenic infections.

## Evidence of Lung Trm Activity Against *Mtb* Infection

Although there are relatively few studies that have directly investigated the role of Trms in TB, the evidence they provide is compelling. In an early study, Connor et al used FTY720 to study the importance of T-cell recruitment from lymph nodes in the recall response to BCG infection. FTY720 is an immunomodulating drug that, like CD69, blocks S1PR activity and thus prevents lymphocytes from exiting lymph nodes via the S1P gradient. Administration of FTY720 during infection but after vaccination (with BCG), was found to have no effect on vaccine efficacy (46), suggesting the vaccine induced T-cells were already present in the lung, although they were not defined at the time as Trm cells. As expected, giving FTY720 prior to vaccination, diminished protection, showing recruitment of vaccine specific T-cells to the lung from secondary lymphoid organs was essential. Mice in this study were all vaccinated sub-cutaneously with no adjuvant, showing that lung Trm formation is possible under these circumstances, although it was unlikely to have been optimal. This is supported by Bull et al., who showed intradermal BCG vaccination in the tail generated long lived TB specific CD4^+^ T-cells in the lung parenchyma expressing high levels of CD69 (108). In this study parenchymal T-cells were identified using intravascular staining, a powerful technique which is discussed in more detail later. Importantly, the same group went on to show that, although intradermal BCG generated TB-specific lung Trm, intranasal vaccination generated them at a higher frequency and was more protective, consistent with suboptimal lung Trm generation via the intradermal vaccination route (109). Again exploiting the properties of FTY720, Florido et al. recently showed that mice intranasally vaccinated with a recombinant influenza A virus vaccine expressing an immunodominant *Mtb* epitope from antigen 85A (PR8.p25), were protected from subsequent infection with *Mtb* when further T-cell recruitment to the lung was blocked (110). Importantly, treatment of unvaccinated mice with FTY720 significantly increased lung mycobacterial burden upon infection, confirming that, in the absence of a pathogen specific T-cell population in the lung, recruitment of newly primed T-cells from circulation is required. In both studies, however, once priming has occurred, further recruitment from circulation is not needed, implying the protective T-cells are already at the site of disease in the lung.

Rather than blocking peripheral T-cell recruitment as a way of demonstrating the importance of tissue resident T-cells, other groups have sought to specifically boost lung Trm via novel vaccination routes. Intratracheal injection of BCG, for example, has been shown to improve vaccine activity, and reduce lung burden of *Mtb* by nearly 2 logs at the highest dose, compared to sub-cutaneous BCG (111). This was associated with a much higher frequency of TB-specific CD4^+^ T-cells in lung homogenate, although the responding cells were not phenotyped for Trm markers.

Copland et al. sought to enhance Trm formation by boosting BCG vaccinated mice with intranasal application of *Bacillus subtilis* spores coated with TB antigens (112). This approach improved protection over BCG vaccination alone and was associated with an expansion of CD69^+^CD103^+^ Trm cells in the lungs that was not seen with either BCG alone or uncoated spores. Boosting BCG vaccinated mice via intranasal or intratracheal administration of nanoparticles coated with *Mtb* antigens was also found to induce CD69^+^CD103^+^ TB-specific lung Trm compared to subcutaneous BCG alone or uncoated nanoparticle (113). In both studies, the vaccine induced expansion of lung Trms was associated with improved protection from subsequent TB challenge. However, in neither case was the activity of these cells formally tested by adoptive transfer or other approaches. Intranasal boosting with a Sendai virus vectored TB vaccine, SeV85AB, was also found to elicit antigen-specific CD103^+^ CD4^+^ and CD8^+^ T-cell responses in the lung and was associated with a reduction of mycobacterial load in lung and spleen compared to subcutaneous BCG alone (114). Interestingly, in this case protection was exclusively mediated by CD8^+^ T-cells and was lost when these cells were depleted, which was not the case in the BCG vaccinated group.

Perdomo et al. found that both intratracheal and intranasal administration of BCG enhanced the generation of both CD4^+^ and CD8^+^ CD69^+^ Trms, that expressed high levels of CD103 and CXCR3, and improved protection against subsequent *Mtb* challenge, compared to subcutaneous BCG (21). These authors went a step further, by adoptively transferring airway T-cells, via intratracheal injection, into naïve mice, and demonstrating they mediate protection compared to control mice receiving only Phosphate Buffered Saline. However, these findings are complicated by the fact that transferred cells were derived from BAL fluid rather than lung parenchyma and that the protection offered by cells with a CD69^+^ Trm phenotype, was not different from those lacking this marker. In addition, the authors did not test T-cells from either unvaccinated or subcutaneous BCG vaccinated mice by adoptive transfer to demonstrate any superiority of Trms generated by airway vaccination.

Novel vaccine candidates have also been used in the mouse model to generate lung homing T-cells. Using a prime boost vaccination approach, Woodworth et al. observed that subcutaneous vaccination three times with a cationic adjuvanted protein-based TB vaccine (H56/CAF01) generated circulating CD4^+^ T-cells with accelerated lung-homing properties post-*Mtb* exposure (93). In this study, H56 vaccinated mice had low mycobacterial burden in the lungs, and TB-specific CD4^+^ T-cells in the lung were multifunctional.

All the above studies were done in mice, which, although highly informative, lack many of the features of human pulmonary TB infection, including the generation of caseating lung granuloma. Non-Human Primate (NHP) models are generally considered to more closely reflect the pathology of human TB, but studying Trm in these animals comes with additional challenges. Intravenous injection of BCG in NHPs has been shown by several groups to provide superior protection to standard intradermal inoculation (115), and surprisingly, vaccination by this route can boost lung Trm responses (116). Others have used mucosal vaccination with BCG or subunit vaccines to significantly boost TB-specific lung T-cell responses (117, 118) with better disease outcome following *Mtb* challenge due more to a reduction in lung damage (119). Kaushal et al. examined the effect of mucosal vaccination with BCG or the attenuated SigH *Mtb* mutant vaccine strain (120). Interestingly, in their hands BCG had little impact on the lung T-cell response and only offered minor protection, however, the SigH mutant greatly boosted lung T-cell responses and induced the formation of inducible bronchioalveolar lymphoid tissue (iBALT) thought to be protective in TB infection (94, 95). Importantly, this was associated not only with reduced lung pathology, but a significant reduction in lung mycobacterial burden and survival of all animals from an otherwise lethal infection (120). More recently, mucosal vaccination with BCG, but not intradermal, was found to protect NHP from repeated low dose exposure through the induction of IL-17 producing T-cell response in the airway (22). Taken together these studies in mice and NHP strongly imply that TB vaccination strategies which induces lung or airway Trm are significantly more protective than those that do not.

As mentioned, one of the key challenges in these experiments is distinguishing parenchymal Trm cells from contaminating PBMCs in lung homogenate (35). Sakai et al. directly addressed this issue through the injection of fluorescently labeled anti-CD45 antibody immediately prior to removing the lungs (19). By this methodology, developed by Anderson et al. blood leukocytes are labeled but tissue penetration is limited (42, 43). This system was then used to study the activity of lung resident T-cells by aerosol infecting mice with *Mtb*, removing their lungs at 30 days and FACS sorting the parenchymal and blood T-cells from lung homogenate based on the presence of anti-CD45 fluorescent antibody. Adoptive transfer of these cells, to naïve, TCRα^−/−^ transgenic mice, prior to *Mtb* infection, demonstrated parenchymal T-cells, which expressed the Trm CXCR3^+^KLRG1^−^ phenotype, had far greater activity than circulating T-cells (18-fold vs. 4-fold reduction in lung bacterial burden) (19). This observation was confirmed in a follow-up study from the same group, where it was found that IFN-gamma production by CXCR3^+^KLRG1^−^ lung T-cells was not a major component of this protective effect (60). Indeed, expression of the inhibitory T-cell surface molecule PD-1 was critical to limit IFN-gamma production and prevent lethal lung pathology. This is supported by a recent elegant vaccination study showing that protective lung Trm with a KLRG1^−^PD1^+^ phenotype is generated by mucosal vaccination with BCG, but not by intradermal vaccination (109). The potential importance of PD-1 expression highlights the fact that Trm activity, though associated with a protective effect, can have pathological consequences when not regulated. Parallel observations were made by Moguche et al. who found that TB specific CXCR3^+^KLRG1^−^PD1^+^ CD4^+^ T-cells preferentially homed to the lung parenchyma and rapidly expanded on antigen re-encounter (121). Adoptive transfer of this subset into TCR KO mice resulted in 10-fold greater reduction in lung CFU compared to KLRG1^+^PD1^−^ CD4^+^ T-cells, supporting the superior antimycobacterial activity of the Trm-like subset. Importantly, the authors also demonstrated this subset had characteristics of long-lived memory cells, as adoptively transferred KLRG1^−^PD1^+^ CD4^+^ T-cells persisted in lung parenchyma in the absence of antigen stimulation, whilst the KLRG1^+^ subset did not. In this study, the authors noted intriguing similarities between the protective Trm-like subsets and Follicular Helper T-cells (Tfh), which both appear to rely of the transcription factor BCL6. Torrado et al. also found KLRG1^−^PD1^+^ T-cell preferentially homed to the lung parenchyma and provided superior protection from *Mtb* infection when adoptively transferred to TCR KO mice (122). Interestingly, the protective Trm subset was preferentially generated by knocking out the IL27 receptor, implicating this signaling pathway in inhibiting Trm development. Both studies also highlight an important role for IL-2 in maintaining protective lung Trm.

Although adopting differing approaches, these studies all provide direct experimental evidence of the importance of T-cells that readily enter the lung parenchyma and establish persistent memory populations in controlling *Mtb* infection. Importantly, taken together these data also highlight two key factors; first, that non-protective T-cells phenotypes that do not migrate into the lungs are generated by TB infection, and possibly by vaccination; and second, that vaccine strategy can affect the proportion of Trms cells in the lung. This provides both a potential reason for why circulating TB specific T-cells do not generally correlate with immune protection and hope that human TB vaccine efficacy may be improved through novel vaccination strategies.

## Final Thoughts

As highlighted above, the generation of lung Trms by vaccination depends on a number of factors. TB specific Trms can be induced by initial mucosal vaccine administration (21, 109, 113, 120); as prime boost strategy to improve efficacy of BCG vaccine (112–114) or multiple boosts subcutaneously (93). Furthermore, the vaccine vector can also influence the type of T-cells (CD4^+^ or CD8^+^) that will confer the greatest protective immunity (112–114). The role of Trm in TB protection still needs further experimental confirmation, particularly in NHPs, but induction of this subset has the potential to improve early control of infection and may even provide sterilizing immunity. Finally, and most importantly, we need to determine how our improved understanding of lung Trm can best be incorporated into the development and deployment of vaccines against TB in humans. It is not apparent that any of the current vaccines in the clinical and pre-clinical pipe line were developed with the clear aim of inducing TB-specific lung Trms. However, there may be opportunities to enhance the activities of BCG, or the promising current vaccine candidates. For example, the formation of lung Trm could be enhanced following standard sub-cutaneous vaccination with BCG and/or an alternative, by subsequent administration of nanoparticles, or other agents containing TB antigens via the mucosal route. Furthermore, novel primary vaccination routes could be explored, such as intravenous injection. Whilst this has many technical and safety challenges, the data demonstrating the boosting of lung Trm and increased protection is compelling and hard to ignore. It may be possible to get around safety issues by using killed BCG or other non-live vaccine candidates, which requires further investigation in animal models. In addition, although intravenous vaccination may never be practical on a large scale, it may be possible for targeted treatment of high-risk groups such as TB house hold contacts. Alternatively, by understanding the mechanistic basis underpinning the ability of intravenous vaccination to generate Trms, it may be possible to achieve the same effect via different vaccination routes.

## Author Contributions

PO and JP literature research and writing. AL literature research and editing.

### Conflict of Interest Statement

The authors declare that the research was conducted in the absence of any commercial or financial relationships that could be construed as a potential conflict of interest.
